# Covalently Crosslinked Nanogels: An NMR Study of the Effect of Monomer Reactivity on Composition and Structure

**DOI:** 10.3390/polym11020353

**Published:** 2019-02-18

**Authors:** Pengfei Liu, Charles M. Pearce, Rozalia-Maria Anastasiadi, Marina Resmini, Ana M. Castilla

**Affiliations:** Department of Chemistry and Biochemistry, SBCS, Queen Mary University of London, Mile End Road, London E1 4NS, UK; pengfei.liu@qmul.ac.uk (P.L.); c.pearce@qmul.ac.uk (C.M.P.); r.anastasiadi@qmul.ac.uk (R.-M.A.)

**Keywords:** nanogels, high dilution radical polymerization, polymeric nanoparticles, monomer conversion, hydrogels, NMR study

## Abstract

Covalently crosslinked nanogels are widely explored as drug delivery systems and sensors. Radical polymerization provides a simple, inexpensive, and broadly applicable approach for their preparation, although the random nature of the reaction requires careful study of the final chemical composition. We demonstrate how the different reactivities of the monomers influence the total degree of incorporation into the polymer matrix and the role played by the experimental parameters in maximizing polymerization efficiency. Nanogels based on *N*-isopropylacrylamide, *N*-*n*-propylacrylamide, and acrylamide crosslinked with *N,N’*-methylenebisacrylamide were included in this study, in combination with functional monomers *N*-acryloyl-l-proline, 2-acrylamido-2-methyl-1-propanesulfonic acid, and 4-vinyl-1*H*-imidazole. Total monomer concentration and initiator quantities are determining parameters for maximizing monomer conversions and chemical yields. The results show that the introduction of functional monomers, changes in the chemical structure of the polymerizable unit, and the addition of templating molecules can all have an effect on the polymerization kinetics. This can significantly impact the final composition of the matrices and their chemical structure, which in turn influence the morphology and properties of the nanogels.

## 1. Introduction

Covalently crosslinked polymeric nanogels have shown promise in multiple applications such as drug delivery, sensing, and catalysis [1,2,3,4,5]. These materials combine the properties typical of polymeric nanoparticles, such as a small size and a high surface-to-volume ratio, with a soft character and the ability to form stable colloidal solutions. The choice of functional monomers and crosslinkers and their ratio enables a fine tuning of the physical and chemical properties of the gels [6,7,8,9] and the introduction of stimuli-responsive characteristics [10,11,12,13,14]. The most common approaches to prepare covalently crosslinked nanogels involve post-polymerization crosslinking, performed on reactive polymer side-chains, or crosslinking during polymerization using di- or multi- functional comonomers (crosslinker) [3,15,16]. The latter usually allows for a better control of particle size and properties of the resulting material [9]. When targeting biomedical applications, polymerizations in a homogeneous phase are often preferred where surfactants are not used [4,15]. Controlled solution radical polymerization techniques such as RAFT (reversible addition-fragmentation chain-transfer polymerization) have been used to obtain micro/nanogels [16,17,18,19] with high monomer conversions, controlled chain architectures, and narrow molecular-weight distributions. Among alternative approaches for the preparation of micro/nanogels, high dilution radical polymerization (HDRP) has been successful [16,20,21]. In this method, control of the growing polymer chains is achieved through steric stabilization when the total monomer concentration (C_M_) is reduced below a critical value, which is dependent on factors such as the solvent, initiator, crosslinker, and polymerization temperature. Advantages of this approach include its simplicity, low cost, and applicability to any type of monomer.

There is considerable interest in understanding the relationship between chemical structure and the physical and chemical properties of micro/nanogels. Thus, given the random nature of a radical polymerization, careful monitoring of monomer conversions is required to gain a better insight into the final chemical composition of the matrix. For this reason, detailed studies of monomer reactivities and reaction conditions are essential to ensure that the isolated polymer has a final formulation that is as close as possible to the initial combination of monomers and crosslinker (feed composition). Despite its importance, literature data on radical polymerizations rarely investigate the actual polymer composition and instead limit description to the initial formulation [22,23,24].

Our group has been interested in NIPAM-based nanogels prepared by HDRP for a diverse range of applications, from drug delivery vehicles to sensors, due to their high solubility, colloidal stability, and biocompatibility [25], together with thermoresponsive properties [6,26,27,28]. Recently, we investigated the morphological changes resulting from variations in chemical structure of functional monomers and crosslinker content in a library of NIPAM and other acrylamide-based nanogels [6]. Interestingly, observations made during that research suggested an impact of monomer reactivity on the chemical composition of the polymeric matrices, and we decided to investigate this further.

Here we report our results on the effect of monomer reactivities on conversion rates, evaluated by ^1^H NMR, and the final composition of nanogels and their properties. Our study initially focused on two-monomer polymers, with both monomers containing acrylamide as the reactive unit, and then expanded to three-monomer polymers. We subsequently evaluated the impact of adding a non-polymerizable fourth component (template), and the effect of changing the chemical structure of the polymerizable unit from acrylamide to vinyl.

## 2. Materials and Methods

### 2.1. Materials

All chemicals were used as received unless otherwise stated. *N*-isopropylacrylamide (NIPAM) and 2-acrylamido-2-methyl-1-propanesulfonic acid (AMPS) were purchased from Alfa Aesar (Heysham, UK). Acrylamide (AM), *N,N’*-methylenebisacrylamide (MBA), caffeine, 2-ethylnaphthalene, and 1,2,4,5-tetramethylbenzene were purchased from Sigma Aldrich (Gillingham, UK). 2,2′-azobisisobutyronitrile (AIBN) was purchased from Sigma Aldrich and used after recrystallization from methanol. Imatinib mesylate (ImMes) was purchased from Insight Biotechnology (Wembley, UK). Deuterated DMSO ((CD_3_)_2_SO) was purchased from Goss Scientific (Crewe, UK). *N*-*n*-propylacrylamide (NPAM), *N*-acryloyl-l-proline (A–Pr–OH), and 4-vinyl-1*H*-imidazole (4VI) were prepared following literature procedures [6,29]. Regenerated cellulose dialysis membrane (MWCO 3500 Da, width 34 mm, diameter 22 mm) was purchased from Medicell International Ltd. (London, UK).

### 2.2. Isolation of Imatinib Free Base (Im) from Imatinib Mesylate

Imatinib mesylate (200 mg, 339 μmol) was dissolved in H_2_O (10 mL). Dropwise, 1 M NaOH (360 μL, 356 μmol) was slowly added, and the solution was stirred for 5 min. The aqueous solution was extracted with DCM (4 × 40 mL). The organic phase was dried over MgSO_4_ and the solvent removed to ca. 1/8 of the original volume under reduced pressure. The remaining DCM was removed at atmospheric pressure, yielding imatinib free base as a slightly off-white powder (160 mg, 96%).

### 2.3. General Procedure for the Preparation of Nanogels

Nanogels were prepared by HDRP following a previously reported procedure [21]. Monomers (NIPAM, NPAM, A–Pr–OH, AMPS, AM, 4VI), template (if applicable), and MBA as crosslinker (CL), in different monomer/CL ratios were dissolved in anhydrous DMSO in a Wheaton™ bottle. The volume of the solvent was adjusted to give the appropriate total monomer concentration (C_M_ = 0.5, 1, or 2%). AIBN as initiator (1, 3, 5, or 10%, compared to total moles of double bonds) was then added to the solution (feed compositions for all nanogels were calculated using Equations S1–S4). The Wheaton™ bottle was sealed and the pre-polymerization solution purged with N_2_. The solution was then heated to the appropriate temperature (60 or 70 °C) for 12, 24, or 48 h. The transparent nanogel solution was then dialyzed against deionized water for 3 days with frequent changes (MWCO 3500 Da, diameter 22 mm), before being frozen in liquid N_2_ and lyophilized (LTE scientific Lyotrap) to yield a white fluffy powder. All the nanogels synthesized were stored at room temperature.

### 2.4. General Procedures for the Determination of Monomer Conversions by ^1^H NMR

^1^H NMR spectra were recorded at 298 K using a Bruker HD 400 MHz BBO Probe or AVIII 400 MHz BBO Probe spectrometers. Chemical shifts for ^1^H are reported in parts per million (δ); δ_H_ values are referenced to the residual solvent signal of DMSO-*d_6_* at 2.50 ppm. 1,2,4,5-tetramethylbenzene or 2-ethylnaphthalene were used as internal standards for quantitative ^1^H NMR measurements. Spectra were processed using Bruker Topspin 4.0.1. Polymerization solutions used for the quantification of monomer conversions by ^1^H NMR were prepared on a smaller scale in Wheaton™ bottles following the same procedure as described in Section 2.3, using DMSO-*d_6_* as the solvent. Two marginally different methods have been used for the quantification of monomer conversions.

Method A: Before sealing the Wheaton™ bottle, an internal standard was added to the pre-polymerization solution. A 500 µL aliquot of the mixture was transferred via microsyringe to an NMR tube, and a ^1^H NMR spectrum was recorded (t = 0 spectrum). The Wheaton™ bottle containing the remaining polymerization solution was sealed, purged with N_2_, and heated to an appropriate temperature for an appropriate time. After polymerization, another 500 μL aliquot of the mixture was transferred, and an ^1^H NMR spectrum was recorded.

Method B: Before sealing the Wheaton™ bottle, a 500 µL aliquot of the mixture was transferred via microsyringe to an NMR tube. The remaining polymerization solution was sealed, purged with N_2_, and heated to an appropriate temperature for an appropriate time. After polymerization, another 500 µL aliquot of the mixture was transferred to a different NMR tube. An appropriate volume of an internal standard stock solution in DMSO-*d_6_* was added to each tube and ^1^H NMR spectra were recorded.

In both cases, the ^1^H NMR spectra acquired were phased and integrated identically using an automated procedure. The concentration of monomers and crosslinker in the initial and final polymerization solutions were determined by comparing the intensities of monomer peaks at 6.58 ppm (4-VI), 5.51 ppm (AMPS), 5.55 ppm (NIPAM), 5.57 ppm (NPAM), 6.07 ppm (AM), 6.62 and 6.36 ppm (A–Pro–OH), and 4.56 or 5.63 ppm (MBA), against the intensities of peaks of the internal standard at 6.88 ppm (1,2,4,5-tetramethylbenzene) or 7.70 ppm (2-ethylnaphthalene). ^1^H NMR spectra used to calculate monomer conversions and overall conversions of polymerizations **N29**, **N36**, and **N42** are shown in Figures S1–S3 of the ESI, as representative examples for all ^1^H NMR analyses.

### 2.5. ^1^H NMR Kinetics

To study monomers conversions over time, ^1^H NMR spectra were recorded at different time intervals using a similar procedure to the one outlined in Section 2.4. Polymerization mixtures were prepared in Wheaton™ bottles using DMSO-*d_6_* as the solvent. The pre-polymerization solution was split into 500 µL aliquots and transferred to NMR tubes using a microsyringe. The tubes were sealed with rubber septa, and the aliquots degassed using 3× freeze–pump–thaw cycles before being left under an N_2_ atmosphere. The polymerizations were then initiated at an appropriate temperature for an appropriate amount of time. For quantification of monomers conversion, 1,2,4,5-tetramethylbenzene or 2-ethylnaphthalene were used as NMR internal standards, either by adding it into the pre-polymerization solution or to the post-polymerization mixture before a ^1^H NMR spectrum was recorded.

### 2.6. Dynamic Light Scattering

Nanogels hydrodynamic diameters (*d_h_*) measurements were obtained by dynamic light scattering (DLS) measurements with a Zetasizer Nano ZS (Malvern Instruments Ltd., Malvern, UK) at 25 °C. All measurements were performed in triplicate (at a concentration of 1 mg mL^−1^) after sonicating for 10 min and filtering through a 0.2 μm syringe filter. Particle sizes given for the nanogels represent estimates of the mean hydrodynamic diameter by number. Size distribution is given by intensity, number, and volume for all measurements (Figures S5–S14).

### 2.7. Procedure for the Determination of the Volume Phase Transition Temperatures

Volume phase transition temperatures (VPTTs) were determined by monitoring the changes in optical transmittance (at 500 nm) of the nanogels in deionized water (1 mg mL^−1^), while increasing temperature from 20 to 65 °C (0.5 °C min^−1^), using a Cary 100 UV-Visible spectrometer (Agilent Technologies, Santa Clara, CA, USA), equipped with a temperature controller. The VPTT was determined as the temperature at which there is a 50% loss of transmittance (Figure S15).

## 3. Results and Discussion

**Figure 1:**
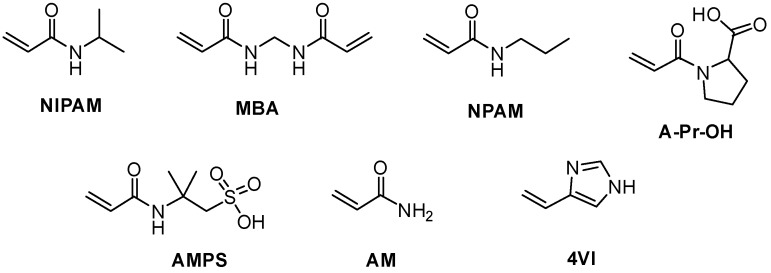
Monomers and crosslinker used in the preparation of nanogels.

Knowing the chemical composition of polymeric matrices obtained via radical polymerization is very important, as it influences all the properties of the material, yet in some cases it can be challenging to obtain, due to the random nature of the process. Previously we have used a combination of monomer conversions, determined by ^1^H NMR, and chemical yields to estimate the chemical nature of different acrylamide-based nanogels obtained by high dilution radical polymerization (HDRP) [6]. In this work, we sought to study how key parameters such as monomer structure, initiator content, total monomer concentration, and temperature can influence the chemical composition and morphology of covalently crosslinked nanogels obtained by HDRP. In order to minimize the number of variables, we chose to carry out all polymerizations in DMSO, to ensure good solubility of all the monomers used, while AIBN was used as the initiator. Figure 1 shows the structures of all the monomers used in this study.

### 3.1. Two-Monomer Nanogels

The first part of the work focused on a series of nanogels based on NIPAM crosslinked with MBA. Small-scale (1–3 mL) polymerizations were performed in deuterated DMSO to determine the monomer conversions by ^1^H NMR analyses of initial and final polymerization solutions. When carrying out NMR-based conversion studies, the quantitative measurements require the use of an internal standard. In the first set of experiments (80 mol % NIPAM and 20 mol % MBA), 2-ethylnaphthalene was used as internal standard and was added either to the pre-polymerization mixture or after polymerization, as described in the experimental section. Both approaches resulted in analogous results, suggesting that the presence of the internal standard in the reaction mixture does not affect the monomer conversions (Table S1). Furthermore, reproducibility studies of the method for calculating monomer conversions by ^1^H NMR revealed an inter-assay variability below 1% (Table S1).

**Figure 2:**
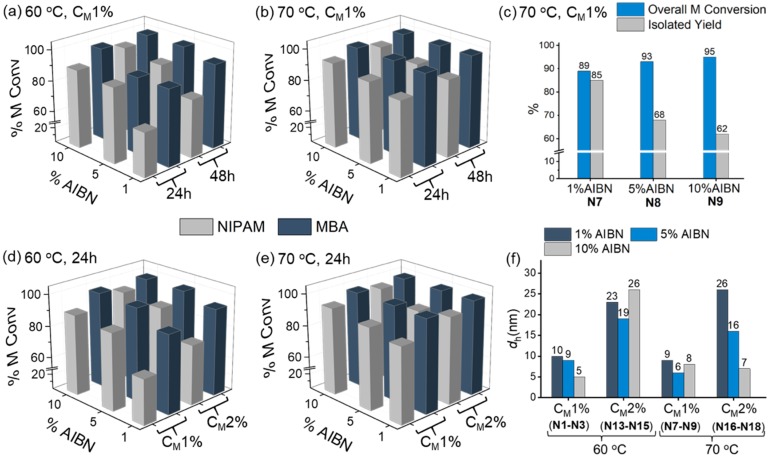
(**a**)–(**e**) Graphs comparing monomer conversions, calculated by ^1^H NMR analyses, for polymerizations of NIPAM (80 mol %) and MBA (20 mol %) carried out under different conditions: (**a**) Nanogels **N1**, **N2**, and **N3** polymerized for 24 h with 1, 5, and 10% AIBN, respectively, and **N4**, **N5**, and **N6** for 48 h with 1, 5, and 10% AIBN, respectively. In all cases C_M_ is 1% and T = 60 °C. (**b**) Nanogels **N7**, **N8**, and **N9** polymerized for 24 h with 1, 5, and 10% AIBN, respectively, and **N10**, **N11**, and **N12** polymerized for 48 h with 1, 5, and 10% AIBN, respectively. In all cases, C_M_ was 1% and T = 70 °C. (**c**) Comparison of overall monomer conversions and isolated yield measured for nanogels **N7–N9**. (**d**) Nanogels **N1**, **N2**, and **N3** polymerized with 1, 5, and 10% AIBN, respectively, and C_M_ was 1%, and **N13**, **N14**, and **N15** with 1, 5, and 10% AIBN, respectively, and C_M_ was 2% (reaction time = 24 h, T = 60 °C). (**e**) Nanogels **N7**, **N8**, and **N9** polymerized with 1, 5, and 10% AIBN, respectively, and C_M_ 1%, and **N16**, **N17**, and **N18** with 1, 5, and 10% AIBN, respectively, and C_M_ was 2% (reaction time = 24 h, T = 70 °C). (**f**) Graph showing the impact of polymerization temperature, initial concentration of AIBN, and total monomer concentration (C_M_) on the particle size determined by DLS measurements for polymerizations of NIPAM (80 mol %) and MBA (20 mol %). Polymerization time is 24 h in all cases. See original raw data in the ESI (Figures S5–S11).

The rates of polymerization are dependent on individual monomer reactivities, which can vary significantly with small changes in their chemical structure. A simple homopolymerization experiment carried out at 70 °C for 12 h (C_M_ = 1%, AIBN = 1%) resulted in 80% conversion for NIPAM, while the crosslinker MBA was quantitatively converted under the same conditions (Table S3). In copolymerizations, rates are not only dependent on individual intrinsic reactivities of each monomer but also on the presence of other monomers and their relative concentrations in the mixture. When 20 mol % MBA was copolymerized for 24 h with 80 mol % NIPAM under the same conditions, MBA conversion was quantitative, whereas NIPAM conversion reached 87% (**N7**, Figure 2a and Table S2), resulting in 89% total monomer conversion. This suggests that the real crosslinker content for **N7** is slightly higher than in the starting solution, resulting in a matrix that is more rigid.

We first focused on screening different reaction conditions for the NIPAM crosslinked with MBA (20 mol %) system, to evaluate the effect of three parameters: 1) initial AIBN concentration (1, 5, and 10 mol %), 2) lower reaction temperature (60 °C), and 3) reaction time (24 and 48 h). The monomer conversions resulting from this screening are summarized in Figure 2 and Table S2. The data on monomer conversions were evaluated together with the chemical yields after isolation of the nanogels. The half-life of the initiator, AIBN, in DMSO increases from 3.9 to 15.9 h when the reaction temperature decreases from 70 to 60 °C [30,31]. We thus aimed to evaluate the impact of a slower initiation reaction on final monomer conversions. For polymerizations at 70 °C, the monomer conversions reached a maximum after 24 h, while for reactions at 60 °C, 48 h were required to reach similar conversion values. Increasing the concentration of initiator used had a larger impact on conversions. At 60 °C, an increase in AIBN initial concentration from 1 to 5% resulted in an increase in NIPAM conversion of 20% at 24 h (**N1**–**N3**) and of 10% at 48 h (**N4**–**N6**). Similar trends were observed for an analogous set of experiments at 70 °C (**N7**–**N12**), albeit the effect is less pronounced since all conversions with 1% AIBN were already above 85%. Regarding the concentration of AIBN, it is interesting to observe that, although a higher concentration of initiator resulted in higher monomer conversions, there was a remarkable effect on the chemical yield, which dropped from 85 to 62%, as shown in Figure 2c. This is due to the formation of larger quantities of low M_r_ polymer chains, due to a higher concentration of radicals at any time in the mixture, which were then lost during purification of the nanogels via dialysis, using membranes with a molecular weight cut-off of 3500 Da.

Next, the impact of the total monomer concentration (C_M_) was investigated (Figure 2d,e). Polymerizations were conducted with AIBN concentrations ranging from 1 to 10%, at 60 or 70 °C for 24 h and with C_M_ = 1 or 2%. As expected, monomer conversions increased when the total monomer concentration was doubled, with the effect being particularly noticeable at 1% AIBN, where the increase in monomer concentration results in a 10% increase of the total monomer conversion. These results show that experimental conditions have a very significant effect on the chemical composition of the final polymer matrix and therefore will also influence the various morphological aspects, such as particle size. To evaluate this, the hydrodynamic diameters (*d_h_*) of NIPAM-MBA (20 mol %) nanogels polymerized for 24 h under different conditions of temperature, total monomer concentration, and percentage of initiator were obtained via dynamic light scattering (DLS) measurements (Figure 2f, Table S2). When a 1% total monomer concentration was used, all nanogels (**N1**–**N3** and **N7**–**N9**) showed very similar diameters, within the range of 5–10 nm, suggesting that neither the polymerization temperature nor the AIBN concentration used have an influencing effect. However, when C_M_ was doubled to 2% (**N13**–**N18**), the particle size slightly increased (16–26 nm), possibly due to an increased degree of intermolecular crosslinking given the higher monomer concentration [32]. A larger particle size can lead to a less stable colloidal solution [33], as confirmed by the VPTT values obtained for nanogels **N13**–**N18** via UV-vis measurements (Table S2). VPTT values for nanogels prepared at higher C_M_ (2%), with larger particle size, were found to be at least 1 °C lower than those prepared at C_M_ = 1% (**N1**–**N3** and **N7**–**N9)**. These data highlight the importance of understanding the actual composition of the polymeric matrices and demonstrate that even small changes can impact the morphology and behavior of the nanoparticles.

As a result of this initial screening, the optimized parameters for the synthesis of NIPAM-based nanogels crosslinked with 20 mol % of MBA were identified as C_M_ = 1% and AIBN = 1%, polymerizing at 70 °C for 24 h. These conditions were then used to evaluate the impact of crosslinker content on monomer conversions (Table 1). NIPAM nanogels **N19** and **N20** (5 and 10 mol % MBA) showed that an increased MBA content led to higher NIPAM conversions and hence higher total monomer conversions. Within the low crosslinker content range (5–20 mol %) particle size did not change: very similar values were measured (4–9 nm) for **N7**, **N19**, and **N20**. The VPPT values however, showed some variations, consistent with previously reported data [6].

Within the two-monomer system, we also investigated how subtle changes in monomer structure could affect the degree of incorporation into the polymer matrix. *N*-*n*-propylacrylamide (NPAM) is a constitutional isomer of NIPAM, also with thermoresponsive characteristics (Figure 1). We have used this monomer for the development of a dual responsive nanogel [6]. Despite the small difference in structure, NPAM displays lower reactivity than NIPAM: under analogous homopolymerization conditions, NPAM reacted to 60% conversion compared to 80% obtained for NIPAM (12 h, C_M_ 1%, AIBN = 1%, 70 °C, Table S2). In the copolymerization of NPAM with MBA, however, the effect was less pronounced. The lower reactivity of NPAM did decrease the total monomer conversion obtained for NPAM nanogels crosslinked with 5 mol % MBA (**N21**) when compared with the NIPAM-based counterpart (**N19**) but only by 4% (Table 1). This effect was supressed when higher amounts of the highly reactive MBA were added to the polymerization mixtures (**N22** and **N23)**. The impact on particle size and VPTT values was found to be similar to that of the NIPAM-based nanogels (Table 2 and Table S4).

### 3.2. Three-Monomer System: Chemical Composition of pH and Temperature-Responsive Nanogels

The development of nanogels for specific applications often requires an additional comonomer (functional monomer), whose structure provides the matrix with a particular function. The presence of this additional monomer may influence the reaction kinetics of the three-monomer system and thus the final chemical composition of the resulting material. Optimization of the reaction conditions to ensure maximum functional monomer and crosslinker incorporation is a critical step prior to evaluating the properties of the nanogels.

We recently reported the synthesis and characterization of a thermo- and pH-responsive nanogel [6], based on PNPAM crosslinked with 10 mol % MBA and including a proline-based monomer (A–Pr–OH, 2.5 mol %) as the functional monomer. Using this preparation, we probed the effect of adding a third component on monomer conversions and nanogel composition. The pH-responsive functional monomer A–Pr–OH was copolymerized with MBA as the crosslinker and NIPAM or NPAM as backbone monomers. In our studies, A–Pr–OH displayed very similar reactivity to that of MBA, both under homopolymerization conditions (Table S3) and in copolymerizations (Table 1), with incorporations reaching 98% in most cases. The high reactivity of A–Pr–OH promoted higher NIPAM and MBA conversions in nanogels **N24**–**N26**, containing 5–20 mol % of MBA, but did not affect the conversions of NPAM-based nanogels (**N27**–**N29**). The high monomer conversions obtained for nanogels **N24**–**N29**, polymerized under the optimized conditions, provided evidence of consistency between initial monomer ratios and final polymer composition. The data obtained suggests that in this system the introduction of a third component in the polymerization solution did not negatively impact monomer conversions and the final composition of the matrix; however, this needs to be evaluated each time, as it greatly depends on the monomer structure.

### 3.3. Three-Monomer Polymers in Combination with a Template Molecule

Particular applications, such as sensing, use nanogels in conjunction with a templating approach, where a non-polymerizable species (template) is added to the polymerization mixture to create specific binding sites within the polymer matrix. The presence of this template can impact the polymerization kinetics in different ways, e.g., by forming a complex with the functional monomer, which may alter its reactivity, or by radical scavenging. Thus, the effect of the template on monomer reactivities needs to be carefully examined, although this is often overlooked [24].

**Figure 3:**
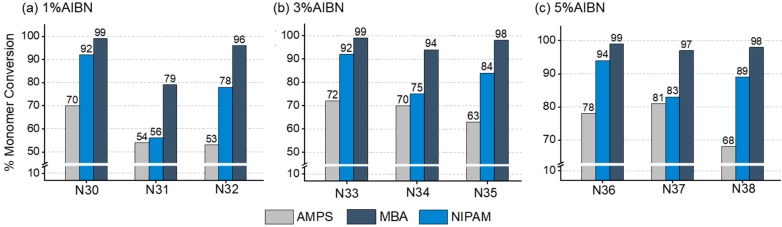
Graphs showing monomer conversions, calculated via ^1^H NMR analyses, for solution polymerizations of AMPS (20 mol %), NIPAM (20 mol %), and MBA (60 mol %) in DMSO-*d_6_*, at C_M_ = 1% and with different AIBN initial concentrations (T = 70 °C, *t* = 24 h): (**a**) 1%; (**b**) 3%; (**c**) 5%. **N30**, **N33**, and **N36** are non-templated nanogels. **N31**, **N34**, and **N37** are templated with imatinib mesylate (1 mol equiv to AMPS). **N32**, **N35**, and **N38** are templated with imatinib free base (1 mol equiv to AMPS). Total monomer conversions are presented in Table S5.

To study how a template molecule can impact polymerization rates we used nanogels developed for the therapeutic drug monitoring of imatinib, a chemotherapeutic agent used in the treatment of chronic myelogenous leukaemia and gastro-intestinal stromal tumors [34,35]. For effective templating of the nanogel, 2-acrylamido-2-methyl-1-propanesulfonic acid (AMPS, Figure 1) was used as the functional monomer. AMPS (20 mol %) was initially polymerized without template in combination with NIPAM (20 mol %) and MBA (60 mol %), under the optimized conditions (C_M_ 1%, AIBN = 1%, 70 °C, 24 h). ^1^H NMR data confirmed quantitative incorporation of MBA and high conversion of NIPAM (>90%), but a lower conversion (70%) was observed for AMPS (**N30**, Figure 3a). Increasing the initial concentration of AIBN to 3 or 5% resulted in an increased AMPS conversion but only by a maximum of 8% (**N36**, Figure 3c). These results suggest that AMPS has a lower reactivity and is the least reactive of the monomers reported here. It is also evident from the homopolymerization of AMPS that the reactivity of this monomer is lower, with a conversion of 36% being achieved after 12 h when using 1% AIBN and C_M_ = 1%, at 70 °C in DMSO (Table S3). As a result of this lower reactivity, the AMPS content in the final polymer is still lower than in the feed composition for the optimized formulation **N36** (16 vs. 20%).

Imatinib is available in two forms: the free base and the mesylate salt. Any possible changes in the polymerization kinetics due to the presence of either compound was evaluated by ^1^H NMR (Figure 3, Table S5). In all cases, AMPS conversions decreased in the presence of the template, with imatinib free base (**N32**, **N35**, and **N38**) appearing to have a more noticeable effect than imatinib mesylate (**N31**, **N34**, and **N37**). This is consistent with the observed decrease in AMPS reactivity (14% conversion) under homopolymerization conditions in the presence of imatinib free base. We ruled out a radical quenching effect by the template, as no degradation of imatinib was observed when heated to 70 °C for 24 h in the presence of AIBN in DMSO (Figure S4). We thus hypothesize that the observed effect of the template is due to the interaction between AMPS and imatinib, resulting in an altered AMPS reactivity to different degrees depending on the use of the free base or the mesylate salt. It is also interesting to note the negative effect on NIPAM conversions when imatinib mesylate is used, possibly due to the presence of the counterion. The results obtained for this system suggest that the presence of a fourth non-polymerizable component in the polymerization mixture has an impact on monomer conversions, resulting in a lower functional monomer content than in the non-templated nanogel. This is particularly relevant and should be investigated carefully when comparisons are drawn between templated and non-templated polymers, such as in the case of molecular-imprinted polymers.

#### The Effect of Introducing a Vinyl Monomer

**Figure 4:**
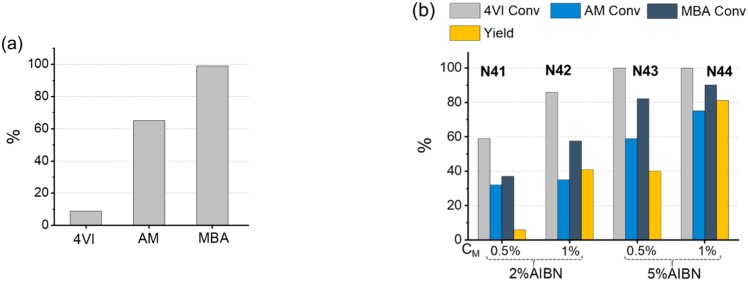
Graphs showing 4VI, AM, and MBA conversions, determined by ^1^H NMR analyses, for (**a**) homopolymerizations in DMSO-*d_6_* (70 °C, 12 h, C_M_ = 1%, AIBN = 1%) and (**b**) copolymerizations in DMSO-*d_6_* (70 °C, 24 h) at two different C_M_ values and using two different AIBN concentrations (**N41**–**N44**). Feed composition in all copolymerizations: 4VI (10 mol %), AM (40 mol %), and MBA (50 mol %). The yellow bar represents the isolated yield.

Often polymerizations require the use of monomers with different polymerizable groups, so we also investigated the effect of these changes on conversion rates. For this purpose, we selected a formulation of nanogels templated with caffeine, containing MBA as the crosslinker, acrylamide (AM) as the backbone monomer, and 4-vinyl-1H-imidazole (4VI, Figure 1), containing a vinyl group as the polymerizable unit, as the functional monomer. Initially, polymerizations were attempted using 4VI (10 mol %), AM (40 mol %), and MBA (50 mol %), under our standard conditions, 70 °C, 24 h, AIBN = 1%, and C_M_ = 0.5 and 1%, but no significant amount of polymer could be isolated. Under homopolymerization conditions (Figure 4a), AM displayed similar reactivity to the acrylamide-based monomers NIPAM and NPAM, while 4VI reacted considerably less, which still did not explain the very low yields. We hypothesized a radical quenching effect by 4VI and thus increased AIBN initial concentration to 2 and 5%, using both C_M_ = 0.5 and 1% (**N41**–**N44**, Figure 4b). The data demonstrate that the increase in AIBN concentration leads to higher monomer conversions, while the higher C_M_ maximized chemical yields. In the preparation of **N44** (5% AIBN and 1% C_M_), monomer conversions above 80% and an 80% chemical yield were achieved.

We further explored the effect of 4VI in a series of copolymerizations with fixed crosslinker MBA content (20 mol %) and varying percentages of 4VI, AM, and NIPAM. Individual and total monomer conversions determined by ^1^H NMR (Table 2), using the optimized conditions for the system (70 °C, 24 h, C_M_ = 1%, and AIBN = 5%), demonstrate that, when 4VI is present in large quantities, such as in **N46**, its quenching effect significantly impacts the overall reaction: even with 5% AIBN, MBA conversion is limited to 31% and results in a total monomer conversion as low as 36%. When 4VI is added in a lower concentration (10 mol %) to NIPAM (**N47**) and AM (**N48**) formulations, the high AIBN percentage (5%) suppresses the inhibition effect more efficiently, resulting in quantitative conversions for 4VI and higher conversions for the backbone monomers (53–60%) and the crosslinker (73–83%). However, these are still lower than conversions obtained in the absence of the vinyl monomer in **N8** and **N45**: 91% (NIPAM), 87% (AM), and >96% (MBA). In agreement with the homopolymerization studies, AM showed a similar reactivity to NIPAM in two-monomer (**N8** and **N46**) and three-monomer (**N47** and **N48**) systems, which allowed us to infer that the change in the chemical structure of the backbone monomer did not have a significant impact on monomer conversions.

The data demonstrate that, regarding monomers with different polymerizable groups, additional studies are required to evaluate their effect on monomer conversions. Any particular quenching factor may require adjustment of the experimental conditions, including the percentages of the individual monomers in the feeding mixture. Interestingly with this specific preparation, when caffeine was added to the polymerization mixture as a template molecule, monomer conversions were not affected. Conversion values for polymerizations in the presence of caffeine (1 mol equivalent with respect to the concentration of 4VI) were analogous to the values obtained for polymerization in its absence (Table S6). The different outcome compared to the previous example with imatinib again highlights the need for more in-depth studies and optimization of radical polymerizations, before conclusions regarding the structure of the polymers and their behaviors can be drawn.

### 3.4. Kinetic Study of Selected Two- and Three-Monomer systems

**Figure 5:**
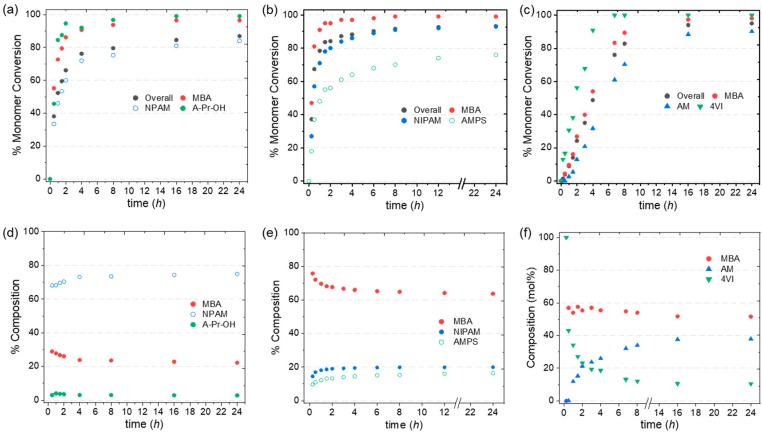
(**a**)–(**c**) Kinetic profiles for nanogels **N29**, **N36**, and **N44**, respectively; (**d**)–(**f**) variation of polymer composition over time derived from 5(a)–(c). [**N29**: 2.5 mol % A–Pr–OH, 77.5 mol % NPAM, and 20 mol % MBA; **N36**: 20 mol % AMPS, 20 mol % NIPAM, and 60 mol % MBA; **N44**: 10 mol % 4VI, 40 mol % AM, and 50 mol % MBA].

**Figure 6:**
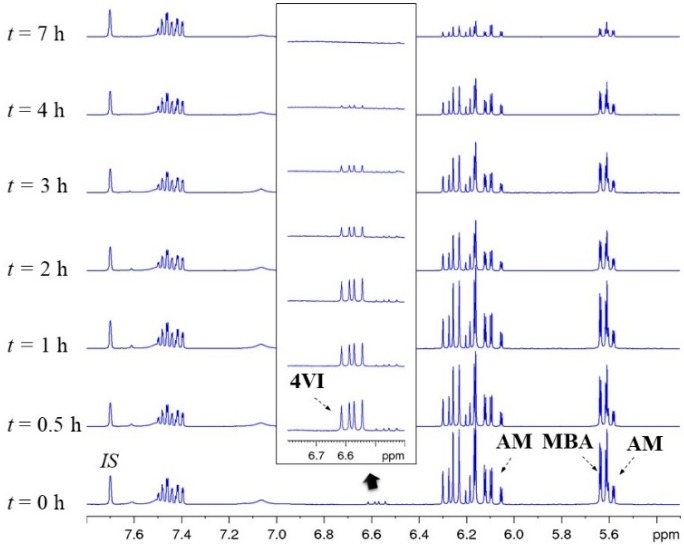
Stacked plot of a selection of the ^1^H NMR (400 MHz, 298 K, DMSO-*d_6_*) spectra recorded at different reaction times during the kinetic study of polymerization **N44** (C_M_ = 1%, AIBN = 5%, 70 °C). Peaks monitored for each monomer and the internal standard (2-ethylnaphtalene) are indicated. Inset: zoomed-in region for the 4VI signal being monitored.

To further probe the impact of monomer reactivity on the polymers chemical structure, kinetic studies were conducted for selected formulations. The monomer conversion was monitored over time for nanogels **N7** (80 mol % NIPAM and 20 mol % MBA), **N26** and **N29** (2.5 mol % A–Pr–OH, 77.5 mol % NIPAM or NPAM and 20 mol % MBA), **N36** (AMPS formulation with 5% AIBN) and **N44** (4VI formulation with 5% AIBN and C_M_ 1%). The data obtained also provide information on how the nanogel composition varies with time. The key kinetic profiles for **N29**, **N36**, and **N44** are shown in Figure 5, while additional ones are presented in the ESI (Figures S16–S18). A stacked plot of the NMR spectra recorded for the kinetic study of polymerization **N44** is shown in Figure 6 as a representative example. The decrease in intensity with time of the peaks at 6.58 ppm (4VI), 6.07 ppm (AM), and 5.63 ppm (MBA) confirm the decrease in concentration of polymerizable units in the solution.

The kinetic profile for the two-monomer system **N7** confirms the faster kinetics of MBA compared to NIPAM (Figure S16), which results in nanoparticles with a more crosslinked core [33,36]. The profiles for nanogels **N26** and **N29** display a similar behavior but with a higher reactivity of A–Pr–OH, resulting in the functional monomer being completely incorporated in the first 2 h of polymerization (Figure 5d and Figure S17). The AMPS formulation (**N36**, Figure 5b) exhibited fast kinetics for both MBA and NIPAM as a result of higher MBA concentration (60 mol %) and AIBN concentration (5%). After 30 min, total monomer conversion had already reached 67% (57% NIPAM and 81% MBA), while AMPS had only reached 37% conversion. Thus, the nanogel is expected to have a higher degree of crosslinking in the core with a higher proportion of the functional monomer AMPS found on the nanogel outer layer (Figure 5e). Interestingly, the kinetic profile of **N44** (Figure 5c) showed a very fast polymerization for the functional unit 4VI, which reached 90% conversion in 4 h, compared to 27% and 12% for AM and MBA, respectively. As shown in Figure 5f, this results in polymer chains being initially enriched in 4VI (up to the first 4 h of reaction) and becoming more enriched in AM as polymerization continues after that time. The results suggest that the particles of nanogel **N44** are more homogeneously crosslinked than the nanogels discussed above, with the functional monomer mainly located in the core of the structure.

## 4. Conclusions

We used ^1^H NMR to determine monomer conversions in high dilution radical polymerizations for the preparation of a number of nanogels based on NIPAM, NPAM, or AM crosslinked with MBA. A study of the polymerization parameters demonstrated their relevance in maximizing conversions and chemical yields, with the total monomer concentration (C_M_) and AIBN percentage being the most significant parameters. The presence and concentration of an additional (functional) monomer can alter the monomer rates of incorporation and thus require careful evaluation before conclusions about the nanogel properties related to chemical composition can be drawn. For example, the use of a highly reactive monomer such as A–Pr–OH promoted higher NIPAM and MBA conversions and ensured a functional monomer content close to the initial formulation. In contrast, AMPS exhibited a much lower reactivity, requiring an increase of the AIBN initial concentration to 5% to achieve acceptable conversions and an optimized functional monomer content. Studying the influence of functional monomers with a different reactive group on the polymerization can be particularly relevant, for example, to identify any quenching effects such as the one shown by 4VI. Similarly, the addition of template molecules for the purpose of creating specific binding cavities, requires a detailed study of its effects on the polymer structure and composition, which is particularly relevant, for instance, when comparing templated and non-templated polymers.

These results provide evidence that the monomer rates of incorporation not only have an important effect on the final composition of the matrix but also on its actual structure: the density of the crosslinked core and the distribution of the functional monomer in the nanoparticles can be significantly varied. This work highlights the importance of carefully studying radical polymerization reactions to understand the composition of the matrices prior to comparing and/or attributing particular properties to a formulation based only on feed composition.

## Figures and Tables

**Table 1:** Monomer conversions and volume phase transition temperatures (VPTTs) for nanogels (NGs) **N7**, **N19**–**N29**.

NG No.	Feed Composition				Monomer Conversion ^1^					
	NIPAM	NPAM	APrOH	MBA	NIPAM	NPAM	APrOH	MBA	*C* ^2^	VPTT ^3^
	mol % Monomer			mol % CL	%			%	%	°C
**N19**	95	0	0	5	81			82	81	37
**N20**	90	0	0	10	83			95	84	41
**N7**	80	0	0	20	87			98	89	39
**N21**	0	95	0	5		77		87	77	27
**N22**	0	90	0	10		83		94	84	32
**N23**	0	80	0	20		86		97	88	34
**N24**	92.5	0	2.5	5	86		>99	86	86	42
**N25**	87.5	0	2.5	10	88		>99	94	89	48
**N26**	77.5	0	2.5	20	90		>99	97	92	58
**N27**	0	92.5	2.5	5		71	90	85	72	29
**N28**	0	87.5	2.5	10		81	98	97	83	34
**N29**	0	77.5	2.5	20		85	98	98	88	40

Polymerization conditions in all cases: C_M_ = 1%, AIBN = 1%, T = 70 °C, 24 h. ^1^ Monomer conversions calculated by ^1^H NMR analyses of initial and final polymerization mixtures. ^2^ *C* is total monomer conversion. ^3^ Volume phase transition temperature measured at a concentration of 1 mg mL^−1^ in deionized water.

**Table 2:** Monomer conversions measured for nanogels **N8** and **N45**–**N48**.

NG No.		Feed Composition			Monomer Conversion ^1^				
	4VI	AM	NIPAM	MBA	4VI	AM	NIPAM	MBA	*C* ^2^
	mol % monomer			mol % CL	%			%	%
**N8 ^3^**	0	0	80	20	-	-	91	99	93
**N45**	0	80	0	20	-	87	-	96	89
**N46**	80	0	0	20	37	-	-	31	36
**N47**	10	0	70	20	98	-	53	77	70
**N48**	10	70	0	20	100	60	-	83	76

^1^ Monomer conversions calculated by ^1^H NMR analysis of initial and final polymerization mixtures. ^2^ *C* is total monomer conversion. ^3^ Data for nanogel **N8** are reported in Figure 2 and Table S2 and included here for comparison. Polymerization conditions: C_M_ = 1%, AIBN = 5%, T = 70 °C, 24 h in DMSO-*d_6_.*

## Supplementary Materials

The following are available online at https://www.mdpi.com/2073-4360/11/2/353/s1. Equations S1–S4 used to calculate feed composition for all nanogels. Table S1: monomer conversion data obtained from six repeats of the polymerization of NIPAM (80 mol %) and MBA (20 mol %) in DMSO-*d_6_*; Table S2: characterization data and monomer conversions and chemical yield data obtained from high dilution radical polymerizations of NIPAM (80 mol %) and MBA (20 mol %) in DMSO(-*d_6_*); Table S3: monomer conversions calculated for the homopolymerization of each of the monomers used; Table S4: chemical yield and DLS data for nanogels **N19**–**N30**; Table S5: monomer conversion and chemical yield data for nanogels **N30**–**N40**; Table S6: monomer conversions, chemical yield, and particle size data for nanogels **N41**–**N44**; Figure S1: partial ^1^H NMR (400 MHz, 298 K, DMSO-*d_6_*) spectra of the polymerization mixtures for the preparation of nanogel **N29** as a representative example for all polymerizations with A–Pr–OH; Figure S2: partial ^1^H NMR (400 MHz, 298 K, DMSO-*d_6_*) spectra of the polymerization mixtures for the preparation of nanogel **N36** as a representative example for all polymerizations with AMPS; Figure S3: partial ^1^H NMR (400 MHz, 298 K, DMSO-*d_6_*) spectra of the polymerization mixtures for the preparation of nanogel **N42** as a representative example for all polymerizations with 4VI; Figure S4: ^1^H NMR (400 MHz, 298 K, DMSO-*d_6_*) spectra of a mixture of imatinib free base and AIBN in DMSO-*d_6_*; Figures S5–S14: dynamic light scattering of nanogels **N1**–**N3**, **N7**–**N9**, and **N13**–**N29**; Figure S15: transmittance change with increasing temperature for nanogels **N1**–**N3**, **N7**–**N9**, and **N13**–**N29**; Figure S16: representations of the kinetic profile for polymerization **N7**; Figure S17: representations of the kinetic profile for polymerization **N26**; Figure S18: representation of the changes in weighted monomer conversions with time corresponding to the kinetic profiles for polymerizations **N29**, **N36**, and **N44**.
