# Supplementary material for: Covalently Crosslinked Nanogels: An NMR Study of the Effect of Monomer Reactivity on Composition and Structure
Source: Polymers (Basel). 2019 Feb 18;11(2):353. doi: 10.3390/polym11020353 (PMC6419204; doi:10.3390/polym11020353)
Supplement: Supplementary file 1 [file polymers-11-00353-s001.pdf]

Supporting Information

Covalently crosslinked nanogels: NMR study of the effect of monomer reactivity on composition and strucutre

Pengfei Liu, Charles M. Pearce, Rozalia-Maria Anastasiadi, Marina Resmini* and Ana M. Castilla*

Department of Chemistry and Biochemistry, SBCS, Queen Mary University of London, Mile End Road E1 4NS, London, United Kingdom.

Email: m.resmini@qmul.ac.uk, a.m.castilla@qmul.ac.uk

1. Supporting Tables

Feed compositions for all nanogels: moles (*n*) of each monomer and AIBN, and volume (*V*) of solvent, were calculated using the following equations:

$$n_{comonomer} = n_{monomer} \times \frac{comonomer\ \%}{monomer\ \%} \qquad (Eq.S1)$$

$$n_{MBA} = n_{monomer} \times \frac{MBA\ \%}{monomer\ \%} \qquad (Eq.S2)$$

$$n_{AIBN} = (n_{monomer} + n_{comonomer} + \ 2n_{MBA}) \times AIBN\% \qquad (Eq.S3)$$

$$V_{DMSO} = \left(\frac{mass_{TOTAL\ monomers} \times (1 - C_M)}{C_M}\right) \times \frac{1}{\rho_{DMSO}} \qquad (Eq.S4)$$

where C_M = total monomer concentration (%*w*/*w*)

Table S1. Monomer conversion data obtained from six repeats of the polymerisation of NIPAM (80 mol%) and MBA (20 mol%) in DMSO-d_6 at 70 °C for 24h. 1% of AIBN and a total monomer concentration (C_M) of 1% was used in all cases. These six entries indicate good reproducibility for the nanogel synthesis and calculation of monomer conversions.

Entry	Feed Composition		Monomer Conversion[a]		
	NIPAM	MBA	NIPAM	MBA	Overall
no.	% mol monomer		%		%
**1**[b]	80	20	88.7	98.2	91.0
**2**[b]	80	20	89.7	98.0	91.0
**3**[b]	80	20	88.6	97.9	90.5
**4**[c]	80	20	89.0	98.0	90.8
**5**[d]	80	20	89.0	99.0	91.0
**6**[e]	80	20	87.8	97.5	90.0

[a]Calculated from ^{1}H NMR analyses of initial and final polymerisation mixtures. [b]Experiment conducted by the same researcher. [b,c,d]Experiments conducted by three different researchers. [e]NMR internal standard (2-ethylnaphtalene) added to the pre-polymerization mixture.

Table S2. Characterisation data and monomer conversions and chemical yield data obtained from high dilution radical polymerisations of NIPAM (80 mol%) and MBA (20 mol%) in DMSO(-d_6).

					Monomer Conversion[a]					
**Nanogel**	**AIBN**	**C_M**	**T**	**time**	**NIPAM**	**MBA**	**Overall**	**Y[b]**	**d_h[c](PdI)[d]**	**VPTT[e]**
n°	mol%	% *w*/*w*	°C	h	%	%	%	%	nm	°C
**N1**	1				68	89	72	75	9.8±3.5 (0.239)	38
**N2**	5	1	60	24	88	98	90	68	9.4±1.6 (0.267)	39
**N3**	10				90	99	92	59	5.2±0.5 (0.275)	40
**N4**	1				77	94	80	*n*	*n*	*n*
**N5**	5	1	60	48	92	99	93	*n*	*n*	*n*
**N6**	10				95	99	96	*n*	*n*	*n*
**N7**	1				87	98	89	83	9.3±1.3 (0.261)	39
**N8**	5	1	70	24	91	99	93	68	5.8±1.5 (0.316)	40
**N9**	10				94	99	95	62	7.9±0.5 (0.363)	40
**N10**	1				89	99	91	*n*	*n*	*n*
**N11**	5	1	70	48	92	99	93	*n*	*n*	*n*
**N12**	10				95	99	96	*n*	*n*	*n*
**N13**	1				77	94	80	72	22.5±8.5 (0.251)	36
**N14**	5	2	60	24	93	99	94	70	19.2±8.0 (0.204)	37
**N15**	10				95	99	96	48	25.6±4.5 (0.206)	37
**N16**	1				94	99	95	73	25.6±12.0 (0.239)	36
**N17**	5	2	70	24	91	99	93	68	15.9±7.4 (0.212)	37
**N18**	10				97	99	97	57	6.9±0.8 (0.206)	37

[a]Monomer conversions were calculated from ^{1}H NMR analyses of initial and final polymerisation mixtures. [b]Y is nanogel polymerisation yield. [c]d_h is hydrodynamic diameter by number as calculated by DLS. [d]PdI is polydispersity index. [e]Measured at a concentration 1 mg/mL. n = not measured

Table S3. Monomer conversions calculated for the homopolymerisation of each of the monomers used in this work.

**Monomer**	**Monomer Conversion[a]**
**MBA**	99%
**NIPAM**	80%
**NPAM**	60%
**A-Pr-OH**	98%
**AMPS**	36%
**AMPS+Im[b]**	14%
**4VI**	9%
**AM**	65%

Polymerisation conditions: 12h, 70 °C, DMSO-d_6 and C_M = 1%. [a]Conversions were calculated from ^{1}H NMR analyses of initial and final polymerisation mixtures. [b]In the presence of 1 equivalent of imatinib free base.

Table S4. Chemical yield and DLS data for nanogels **N19** – **N30**

**Nanogel**	**Feed Composition**					
	**NIPAM**	**NPAM**	**MBA**	**A-Pr-OH**	**Y[a]**	***d_h*[b](PdI)[c]**
**n°**	mol%	Mol%	mol%	mol%	%	nm
**N19**	95	0	5	0	82	5.4±0.2(0.5)
**N20**	90	0	10	0	77	4.1±0.2(0.5)
**N21**	0	95	5	0	51	107.2±6.5(0.1)
**N22**	0	90	10	0	61	6.2±2.1(0.3)
**N23**	0	80	20	0	75	6.1±1.2(0.6)
**N24**	92.5	0	5	2.5	81	3.0±0.2(0.4)
**N25**	87.5	0	10	2.5	84	2.7±0.4(0.5)
**N26**	77.5	0	20	2.5	95	2.6±0.2(0.8)
**N27**	0	92.5	5	2.5	76	90.4±15(0.2)
**N28**	0	87.5	10	2.5	80	5.4±0.2(1.0)
**N29**	0	77.5	20	2.5	89	4.1±0.2(1.0)

Polymerisation conditions: 24 h, 70 °C, DMSO(-d_6) and C_M = 1%. [a]Y is nanogel polymerisation yield. [b]d_h is hydrodynamic diameter by number as calculated by DLS. [c]PdI is polydispersity index.

Table S5. Monomer conversion and chemical yield data for nanogels **N30** - **N40**

**Nanogel**	**Feed Composition**					**Monomer Conversion[b]**				
	**AMPS**	**NIPAM**	**MBA**	**AIBN**	**AMPS:Im[a]**	**AMPS**	**NIPAM**	**MBA**	**Overall**	**Y[c]**
**n°**	mol%		mol%CL	mol%	mol equiv	%		%	%	%
**N30**					-	70	92	99	92	62
**N31**	20	20	60	1	1:1	54	56	79	70	*n*
**N32**					1:1	53	78	96	84	*n*
**N33**					-	72	92	99	92	75
**N34**	20	20	60	3	1:1	63	84	98	85	*n*
**N35**					1:1	70	75	94	85	*n*
**N36**					-	78	94	99	94	71
**N37**					1:1	81	83	97	91	*n*
**N38**	20	20	60	5	1:1	68	89	98	90	*n*
**N39**					2:1	81	90	99	94	*n*
**N40**					3:1	81	89	99	93	*n*

*Polymerisation conditions: 24 h, 70 °C, DMSO and C_M =1%. [a]**N31**, **N34** and **N437** were templated with imatinib mesylate and **N32**, **N35** and **N38 - N40** with imatinib free base. [b]Conversions were calculated from ^{1}H NMR analyses of initial and final polymerisation mixtures. [c]Y is nanogel polymerisation yield. n = not measured (the yield for templated polymers was not calculated as imatinib could not be completely washed out).*

Table S6. Monomer conversions and chemical yields for nanogels **N41-N44**

	Feed Composition					Monomer Conversion[a]				
**Nanogel**	**4VI**	**AM**	**MBA**	C_M	**AIBN**	**4VI**	**AM**	**MBA**	**Overall**	**Y[b]**
**n°**	mol%		mol%	%	mol%	%		%	%	%
**N41**	10	40	50	0.5	2	59	32	37	37	6
**N41-T[c]**						61	35	38	39	7
**N42**	10	40	50	1	2	86	35	58	52	41
**N43**	10	40	50	0.5	5	100	59	82	75	40
**N43-T[c]**						100	63	82	76	50
**N44**	10	40	50	51	5	100	75	90	85	81

Polymerisation conditions: 70 °C, 24 h in DMSO(-d$_6$). [a]Conversions were calculated from ^{1}H NMR analyses of initial and final polymerisation mixtures. [b]Y is nanogel polymerisation yield. [c]In templated nanogels 1 mol% of caffeine with respect to the amount of 4VI was present in the polymerisation mixture.

2. Supporting Figures

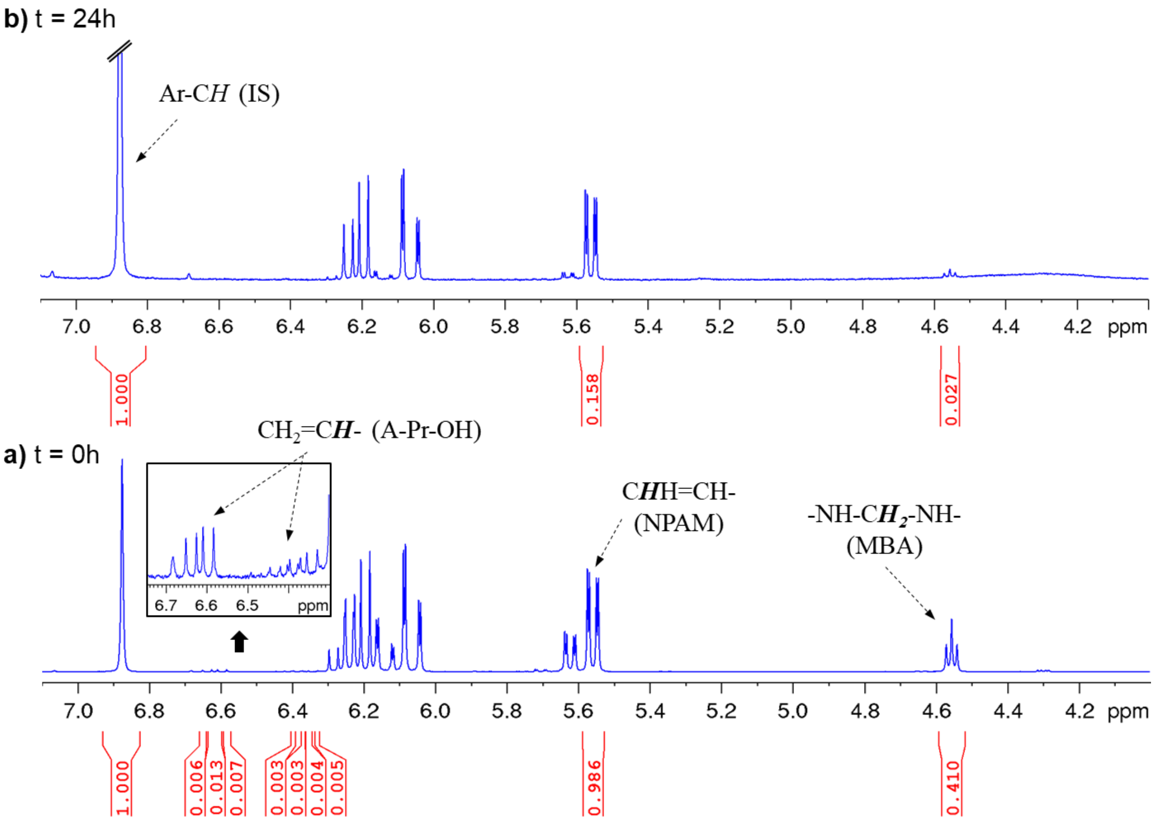

Figure S1. Partial ^{1}H NMR (400 MHz, 298 K, DMSO-d_6) spectra of the polymerisation mixtures for the preparation of nanogel **N29** as a representative example for all polymerisations with A-Pr-OH. a) Spectrum acquired immediately after preparation of the polymerisation mixture, b) spectrum acquired after heating at 70 °C for 24 h. Peaks integrated to calculate conversions (Table 1 and Table S4) are indicated. 1,2,4,5-tetramethylbenzene was used as internal standard (IS). Intensities have been scaled for clarity.

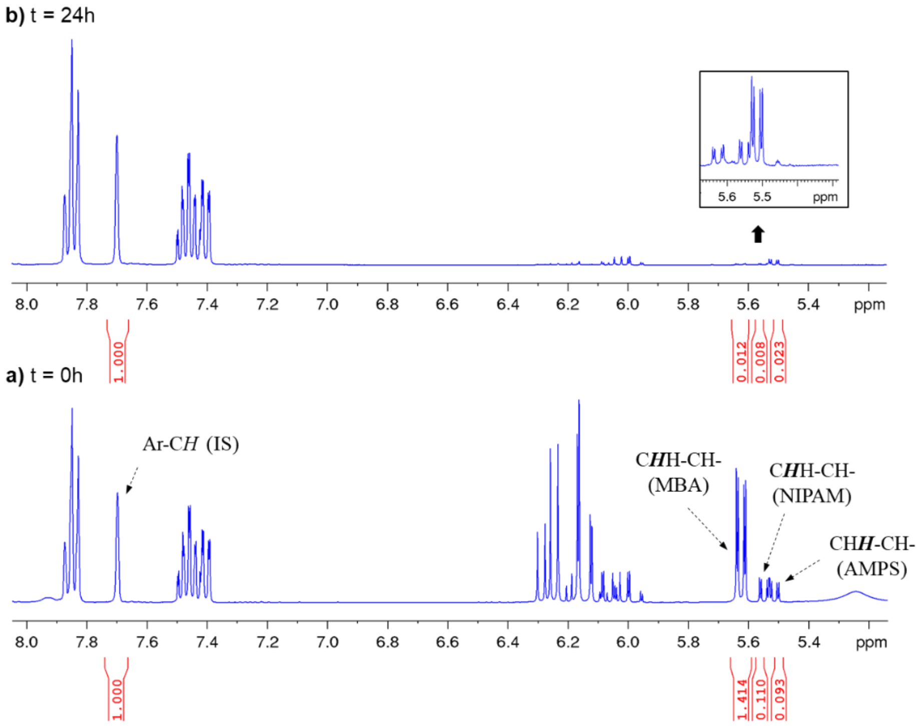

Figure S2. Partial ^{1}H NMR (400 MHz, 298 K, DMSO-d_6) spectra of the polymerisation mixtures for the preparation of nanogel **N36** as a representative example for all polymerisations with AMPS. a) Spectrum acquired immediately after preparation of the polymerisation mixture, b) spectrum acquired after heating at 70 °C for 24 h. Peaks integrated to calculate conversions (Figure 3 and Table S6) are indicated. 2-ethylnaphtalene was used as internal standard (IS). Intensities have been scaled for clarity.

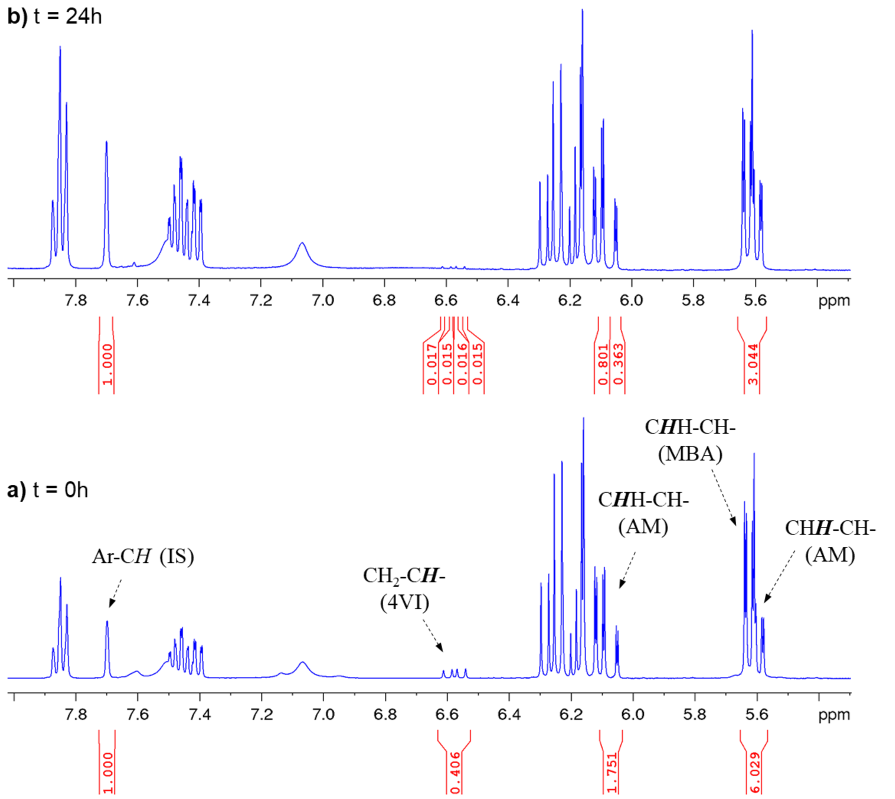

Figure S3. Partial ^{1}H NMR (400 MHz, 298 K, DMSO-d_6) spectra of the polymerisation mixtures for the preparation of nanogel **N42** as a representative example for all polymerisations with 4VI. a) Spectrum acquired immediately after preparation of the polymerisation mixture, b) spectrum acquired after heating at 70 °C for 24 h. Peaks integrated to calculate conversions (Figure 4 and Table S6) are indicated. 2-ethylnaphtalene was used as internal standard (IS). Intensities have been scaled for clarity.

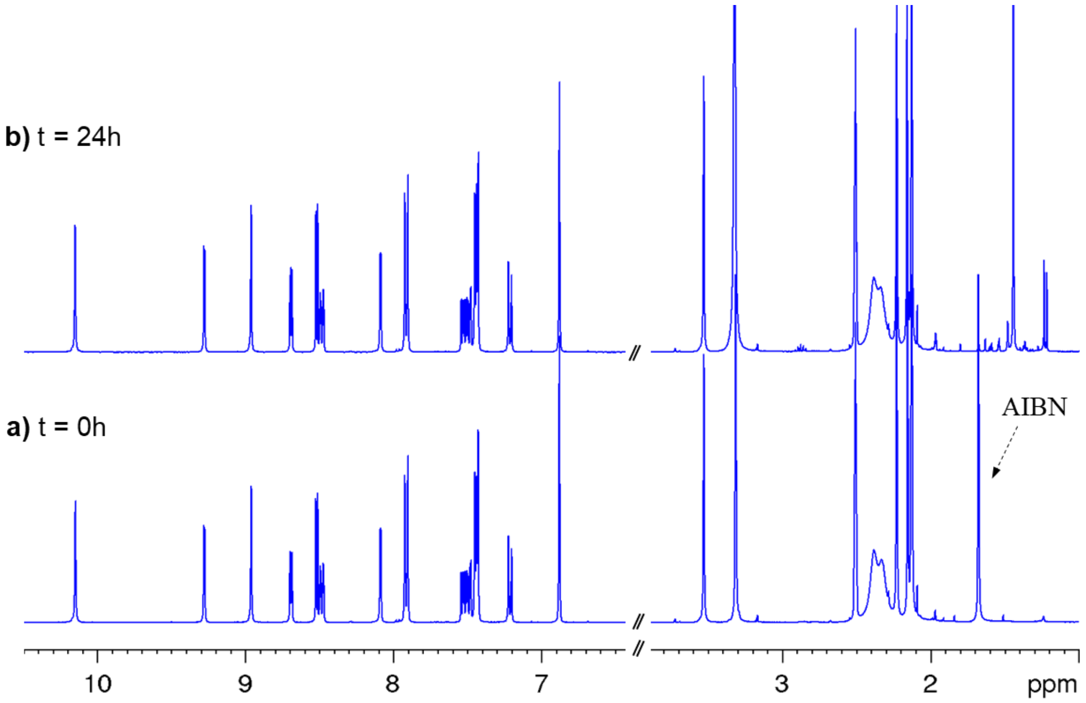

Figure S4. ^{1}H NMR (400 MHz, 298 K, DMSO-*d*$_6$) spectra of a mixture of imatinib free base and AIBN in DMSO-*d*$_6$ **a)** after preparation and **b)** after heating to 70 °C for 24 h. The fact that no new peaks appear on the second spectra show that imatinib does not degrade in the presence of the radicals formed upon AIBN decomposition. Intensities have been scaled for clarity.

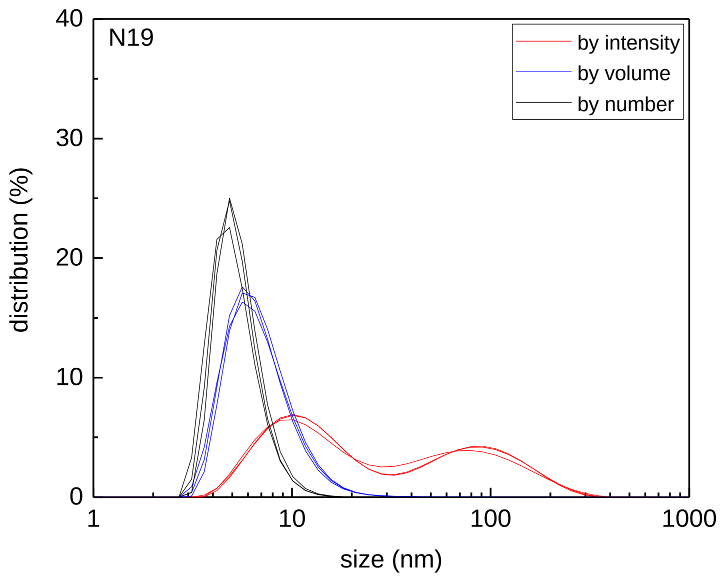

Figure S5. Dynamic light scattering of nanogels **N19**

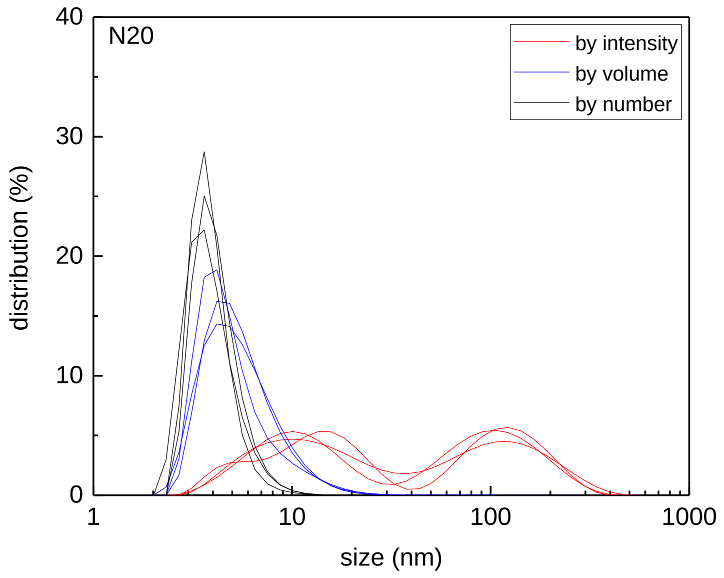

Figure S6. Dynamic light scattering of nanogels **N20**

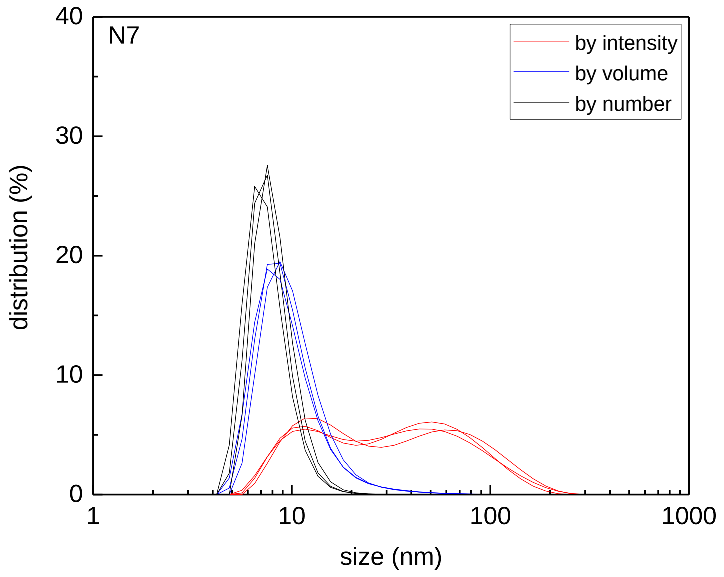

Figure S7. Dynamic light scattering of nanogels **N7**

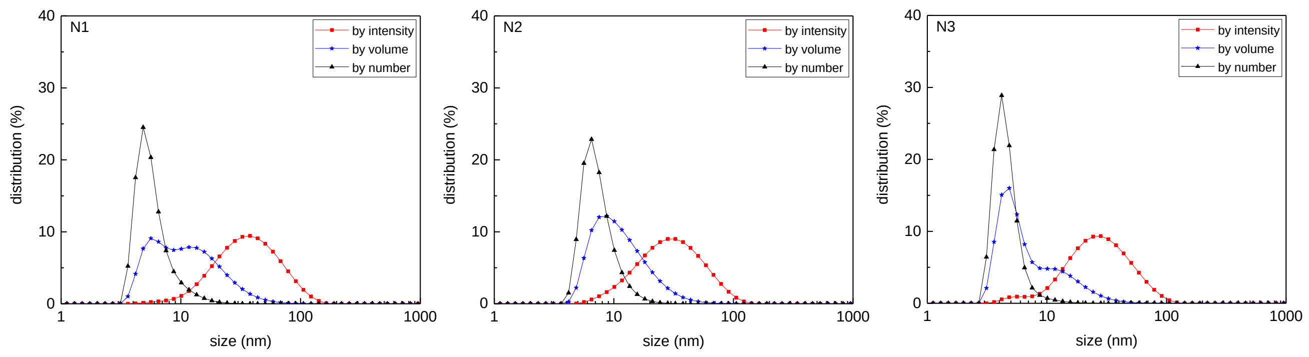

Figure S8. Dynamic light scattering of nanogels **N1**, **N2** and **N3** (measurement were performed in triplicate but only one is shown for clarity)

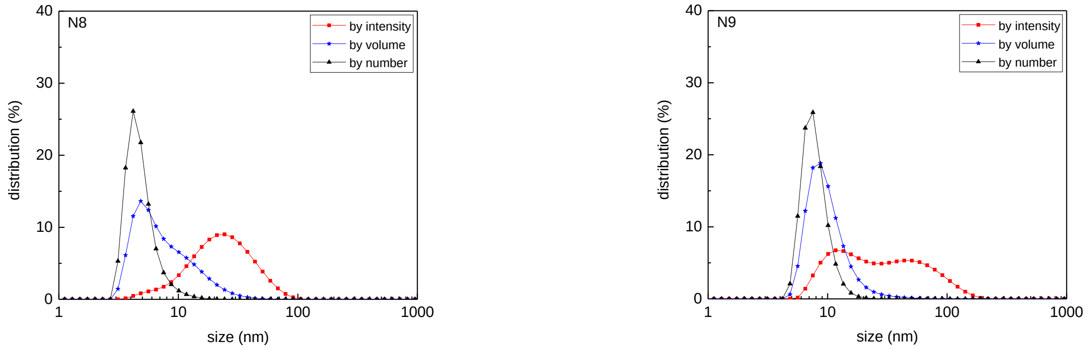

Figure S9. Dynamic light scattering of nanogels **N8** and **N9** (measurement were performed in triplicate but only one is shown for clarity)

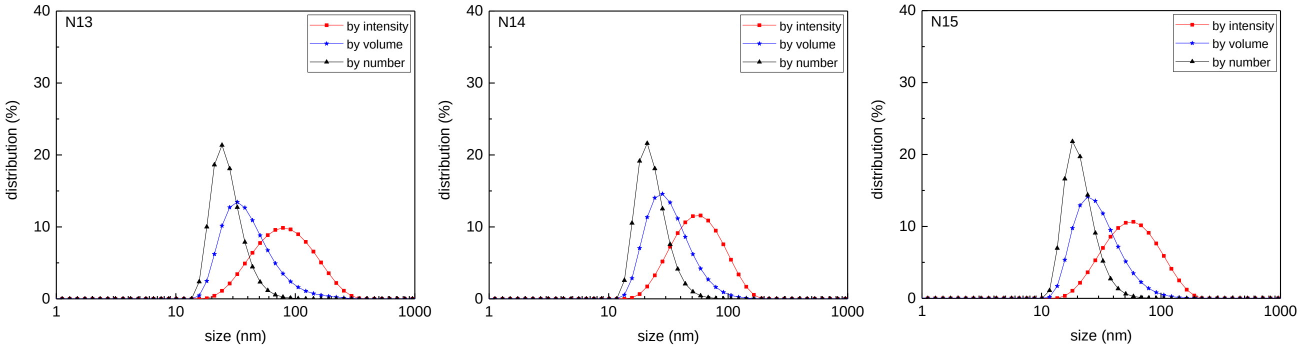

Figure S10. Dynamic light scattering of nanogels **N13**, **N14** and **N15** (measurement were performed in triplicate but only one is shown for clarity)

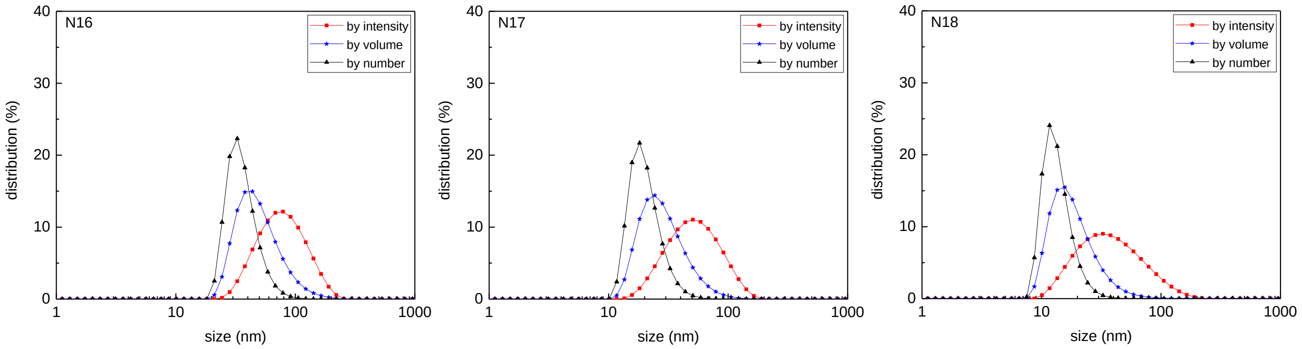

Figure S11. Dynamic light scattering of nanogels **N16**, **N17** and **N18** (measurement were performed in triplicate but only one is shown for clarity)

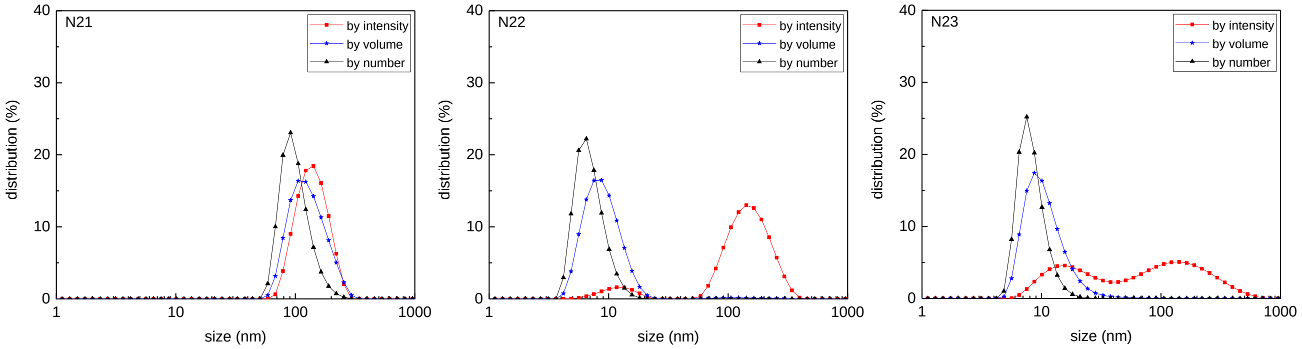

Figure S12. Dynamic light scattering of nanogels **N21**, **N22** and **N23** (measurement were performed in triplicate but only one is shown for clarity)

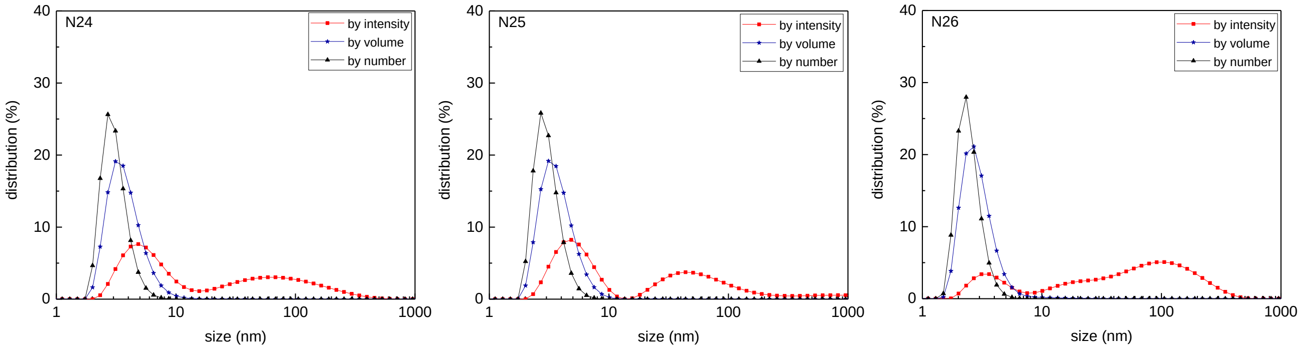

Figure S13. Dynamic light scattering of nanogels **N24**, **N25** and **N26** (measurement were performed in triplicate but only one is shown for clarity)

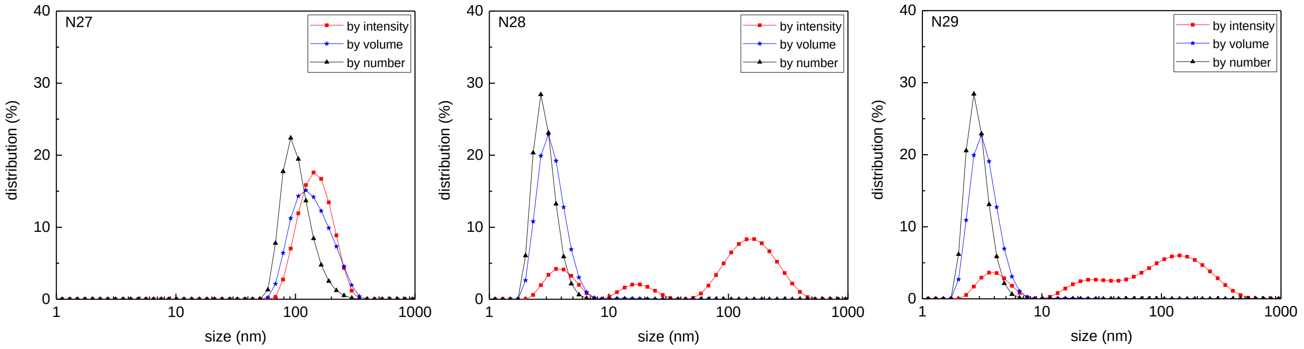

Figure S14. Dynamic light scattering of nanogels **N27**, **N28** and **N29** (measurement were performed in triplicate but only one is shown for clarity)

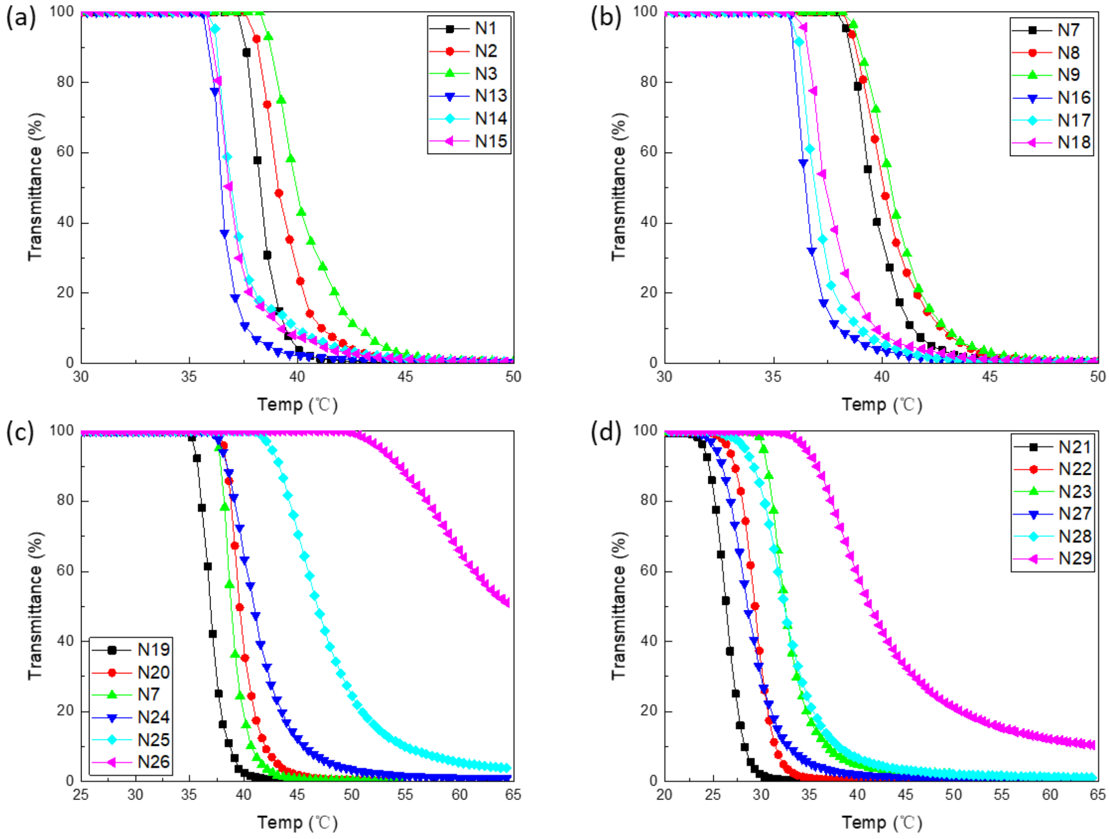

Figure S15. Transmittance change with increasing temperature for nanogels **N1** - **N3**, **N7** - **N9**, **N13** - **N29**. Transmittance was measured at 500 nm at a polymer concentration of 1 mgmL^{-1} in deionised water.

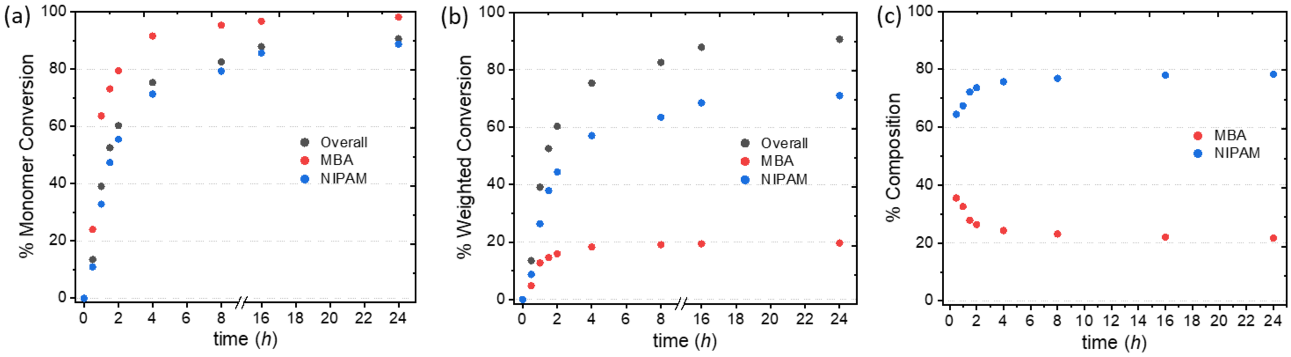

Figure S16. Representations of the kinetic profile for polymerisation **N7**: 80 mol% NIPAM, 20 mol% MBA, C_M = 1%, 1% AIBN, T = 70 ºC, *t* = 24 h. (a) Change in monomer conversions with time; (b) Change in weighted monomer conversions with time; (c) Change in polymer composition with time.

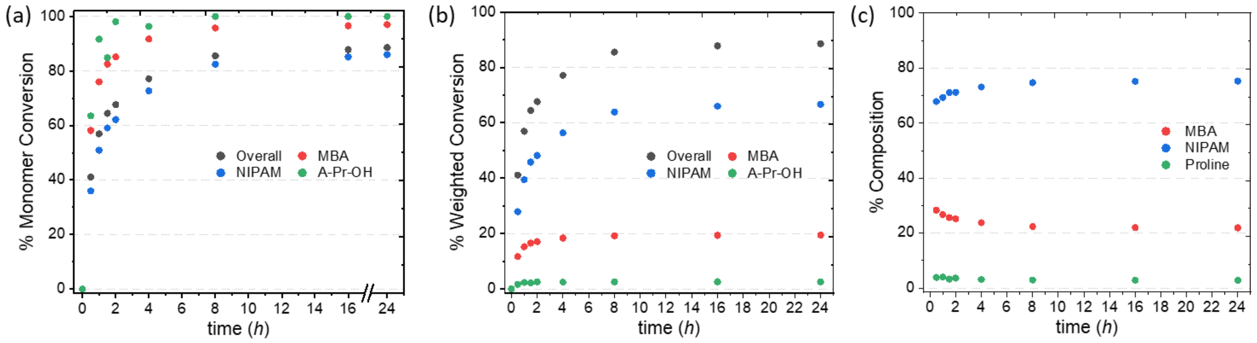

Figure S17. Representations of the kinetic profile for polymerisation **N26**: 2.5 mol% A-Pr-OH, 77.5 mol% NIPAM, 20 mol% MBA, C_M = 1%, 1% AIBN, T = 70 ºC, *t* = 24 h. (a) Change in monomer conversions with time; (b) Change in weighted monomer conversions with time; (c) Change in polymer composition with time.

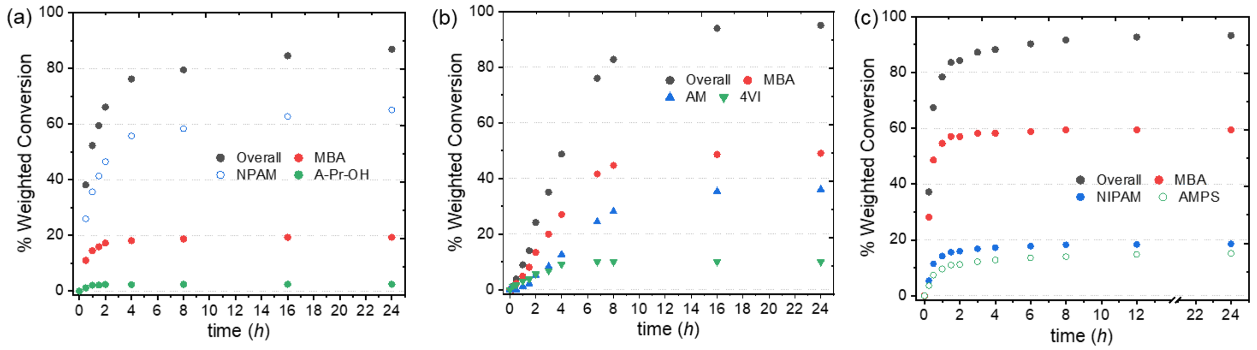

Figure S18. Representation of the changes in weighted monomer conversions with time corresponding to the kinetic profiles for polymerisations **N29** (2.5 mol% A-Pr-OH, 77.5 mol% NPAM and 20 mol% MBA), **N36** (10 mol% 4VI, 40 mol% AM and 50 mol% MBA) and **N44** (20 mol% AMPS, 20 mol% NIPAM and 60 mol% MBA). Polymerisation conditions: C_M = 1%, 1% (**N29**) or 5% (**N36**, **N44**) AIBN, 70 ºC, 24 h.
